# Synthesis, Characterization, and Cytotoxicity Evaluation of Chlorambucil-Functionalized Mesoporous Silica Nanoparticles

**DOI:** 10.3390/pharmaceutics16081086

**Published:** 2024-08-19

**Authors:** Juliana Camila Fischer Karnopp, Juliana Jorge, Jaqueline Rodrigues da Silva, Diego Boldo, Kristiane Fanti Del Pino Santos, Adriana Pereira Duarte, Gustavo Rocha de Castro, Ricardo Bentes de Azevedo, Ariadna Lafourcade Prada, Jesús Rafael Rodríguez Amado, Marco Antonio Utrera Martines

**Affiliations:** 1 Postgraduate Program in Chemistry, Chemistry Institute, Federal University of Mato Grosso do Sul, Campo Grande 79079-900, MS, Brazil; juliana.karnopp@gmail.com (J.C.F.K.); juliana.jorge@proton.me (J.J.); diegoboldo18@gmail.com (D.B.); kristianefdp@gmail.com (K.F.D.P.S.); adriana.duarte@ufms.br (A.P.D.); 2 Postgraduate Program in Nanoscience and Nanotechnology, Biological Science Institute, University of Brasilia, Brasilia 70910-900, DF, Brazil; soljaque@gmail.com (J.R.d.S.); razevedo@unb.br (R.B.d.A.); 3 Postgraduate Program in Environmental Biotechnology, Bioscience Institute, Sao Paulo State University, Botucatu 18618-000, SP, Brazil; gustavo.castro@unesp.br; 4 Postgraduate Program in Biotechnology, Faculty of Pharmacy, Food and Nutrition, Federal University of Mato Grosso do Sul, Campo Grande 79070-900, MS, Brazil; lafourcade.ariadna@ufms.br; 5 Postgraduate Program in Health Sciences, Faculty of Health Sciences, Federal University of Grande Dourados, Dourados 79804-970, MS, Brazil; jesusamado@ufgd.edu.br

**Keywords:** nanocarriers, mesoporous silica, chlorambucil, cytotoxicity, cancer, drug delivery, targeted therapy

## Abstract

This study describes the synthesis and characterization of chlorambucil (CLB)-functionalized mesoporous silica nanoparticles (MSNs) for potential application in cancer therapy. The nanoparticles were designed with a diameter between 20 and 50 nm to optimize cellular uptake and avoid rapid clearance from the bloodstream. The synthesis method involved modifying a previously reported technique to reduce particle size. Successful functionalization with CLB was confirmed through various techniques, including Fourier transform infrared spectroscopy (FTIR) and elemental analysis. The cytotoxicity of the CLB-functionalized nanoparticles (MSN@NH_2_-CLB) was evaluated against human lung adenocarcinoma cells (A549) and colon carcinoma cells (CT26WT). The results suggest significantly higher cytotoxicity of MSN@NH_2_-CLB compared to unbound CLB, with improved selectivity towards cancer cells over normal cells. This suggests that MSN@NH_2_-CLB holds promise as a drug delivery system for targeted cancer therapy.

## 1. Introduction

Cancer is an umbrella term for over 100 diseases characterized by the rapid and uncontrolled proliferation of cells. It has become a global health threat due to its high incidence and mortality rates. Cancer arises from genetic alterations in cells, which occur primarily due to genetic factors (10–20% of cases) or exposure to carcinogenic agents such as smoking, alcohol, physical inactivity, obesity, viruses, and excessive sun exposure (80–90% of cases) [1,2,3]. After cardiovascular diseases, cancer is the second leading cause of death worldwide, with 70% of deaths occurring in low- and middle-income countries [4,5].

Cancer cells divide rapidly and uncontrollably, forming tumors that can spread to other parts of the body through a process called metastasis. Each type of cancer requires a specific therapy; thus, early detection is crucial for an effective treatment [6]. Cancer treatment alternatives include surgery, chemotherapy, radiation therapy, and bone marrow transplantation. In many cases, a combination of these modalities is necessary [7].

**Figure 1:**
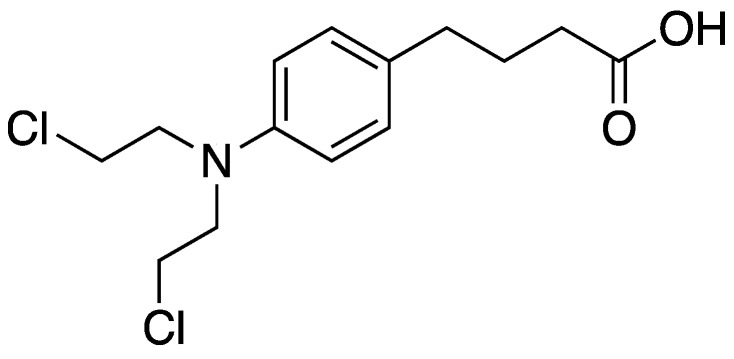
Molecular structure of chlorambucil.

Chemotherapy is considered one of the most powerful tools to combat cancer. The discovery of nitrogen mustard as an alkylating agent in 1942 marked a new era in cancer therapy. Alkylating agents exert their biological activity by binding to DNA, interfering with its replication, and leading to cell death [5]. Chlorambucil (CLB) is one of the pioneering chemotherapeutic agents (Figure 1).

CLB is a drug extensively used for treating patients suffering of lung and colon cancer [5]. Colorectal cancer is the third most common cancer globally, often diagnosed at advanced stages, leading to poor prognosis. On the other side, lung cancer remains a major challenge due to limited treatment options and drug resistance [5,8]. However, CLB has low selectivity, attacking both tumor and normal cancer cells. Consequently, CLB causes diverse and serious side effects such as nausea, vomiting, myeloid leukemia, suppression of blood cell production, alopecia, amenorrhea, and loss of male fertility. [5,8] Additionally, CLB is potentially teratogenic. These facts limit its applications in chemotherapy [8,9]. Therefore, there is a need to develop intelligent drug carriers for CLB to overcome the challenges associated with its clinical application.

Several studies have proposed the use of nanoparticle matrices as drug delivery systems to target high drug loads to the tumor site. In nanoparticles, the drug can be bound or adsorbed to the surface or encapsulated in the core. These forms reduce the risk of a premature drug delivery in the bloodstream, which minimize a possible systemic cytotoxicity. Additionally, inside the core of the nanoparticle, drugs are protected from diverse degradation factors before reaching the target tissue [10,11]. On the other side, nanoparticles improve drug solubility, bioavailability, and tumor specificity. They also can reduce side effects, due to the use of lower drug doses and enhanced treatment effectiveness. Chlorambucil-functionalized nanoparticles could be specifically delivered to cancer cells and complement or replace existing therapies in the future.

Numerous carrier systems have been investigated for drug delivery applications; however, not many medications based on nanoparticles are available in the market [12]. Mesoporous silica, polymeric micelles, drug conjugates, nanogels, liposomes, nanospheres, gold nanoparticles, and chitosan nanoparticles have been employed for this purpose [4,12]. Among the materials that have been used as nanocarriers, mesoporous materials have gained prominence due to their stability, porosity with an ordered structure, high surface area, and large pore volume [13,14]. Within the mesoporous materials, mesoporous silica has emerged as a promising drug delivery carrier due to its non-toxic nature and biocompatibility [15,16,17,18,19]. The ordered mesoporous structure of the silica allows it to be loaded with drugs. On the other side, the presence of silanol groups on mesoporous silica’s surface allow for its functionalization with most diverse drugs [20,21].

Regarding studies on nanoparticles containing CLB for use in drug delivery for cancer treatment, several works are reported in the literature using different CLB loading platforms. For instance, Jiang et al., (2019) [22] reported the development of a catanionic release system to improve chemotherapy treatment strategies. Another recent study, for example, reported the development of folic acid-functionalized graphene oxide particles loaded with CLB. [23] Guo et al. (2020) [24] developed hydrogel peptides containing CLB aimed at improving the efficacy of chemotherapy treatment through controlled release. Iron nanoparticles containing CLB have also been synthesized for use in the treatment/targeting of leukemia [25]. Another example of a study using CLB in loading platforms for controlled release in cancer treatment is the work of Kulig et al., (2022) [26], who developed albumin NPs containing CLB.

However, concerning the use of silica nanoparticles as CLB carriers, the reported studies are scarce. There is the work of Juárez et al., (2022) [27] reporting the development of mesostructured cellular foam containing CLB and the work of Ovejero-Paredes et al., (2022) [28] on the synthesis of fibrous silica particles functionalized with trimethoxysilylpropyl diethylenetriamine and loaded with CLB, folic acid, and a fluorophore agent for potential application as a theragnostic agent.

Driven by these considerations, the present study aimed to synthesize, characterize, and evaluate the cytotoxic activity of mesoporous silica nanoparticles functionalized with CLB for their potential application in cancer therapy.

## 2. Materials and Methods

### 2.1. Chemicals

The chemicals used were cetyltrimethylammonium bromide (CTAB, ≥98%, Sigma Aldrich, São Paulo, Brazil); tetraethyl orthosilicate (TEOS, 98%, Sigma Aldrich); 2,2-azobis(2-methylpropionamidine) dihydrochloride (AIBA, 97%, Sigma Aldrich); l-lysine (≥98%, Sigma Aldrich); styrene (≥99%, Sigma Aldrich); octane (98%, Sigma Aldrich); (3-aminopropyl) triethoxysilane (APTES, ≥99%, Sigma Aldrich); tetrahydrofuran (THF; ≥99.9%, Sigma Aldrich); 3-chloropropyl-triethoxysilane (CPTMS, ≥98%, Sigma Aldrich); sodium hydrate (90%, Sigma Aldrich); europium chloride hexahydrate (EuCl_3_.6H_2_O, ≥99.99%, Sigma Aldrich); 2-thenoyltrifluoroacetone (TTA, ≥99%, Sigma Aldrich); 1,0-phenanthroline monohydrate (1,10-phen, ≥99%, Sigma Aldrich); chlorambucil (≥98%, Sigma Aldrich); isopropyl alcohol (99.5%, Dinâmica, Indaiatuba, Brazil); ethylenediaminetetraacetic (EDTA, ≥99%, Sigma Aldrich); diciclohexilcarbodiimide (DCC, ≥99%, Sigma Aldrich); N-hidroxisuccinimide (NHS, ≥98%, Sigma Aldrich); triethylamine (TEA, ≥99%, Sigma Aldrich); trypan blue (≥99.9%, Sigma Aldrich); phosphate buffer saline (PBS, Sigma Aldrich). Dimethyl sulfoxide (DMSO, 99.9%, J.T Baker); sodium hydroxide (Proquimios, Rio de Janeiro, Brazil); ethanol anhydrous (99.8%, Dinâmica); ninhydrin (≥ 100%, Merck, São Paulo, Brazil); Dulbecco’s Modified Eagle Medium (DMEM, Gibco, Diadema, Brazil) containing 10% fetal bovine serum (FBS, Gibco); Trypsin/EDTA (Gibco); Bovine serum albumin (BSA, Life Technologies, São Paulo, Brazil); penicillin (Life Technologies); streptomycin 100 µg/mL—STR/PEN (Life Technologies); (3-(4,5-dimethylthiazol-2-yl)-2,5-diphenyltetrazolium bromide (MTT, ≥98%, Life Technologies); and nitrogen gas (White Martins, Campo Grande, Brazil). All reagents were of analytical grade and were used as received without further purification; throughout the experiments, Milli-Q grade water (Millipore system) was used.

### 2.2. Synthesis of Mesoporous Silica Nanoparticles (MSNs)

**Scheme 1:**
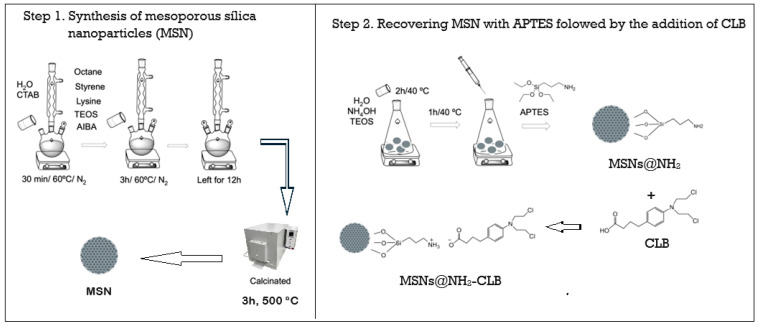
Synthesis of mesoporous silica nanoparticles grafted with chlorambucil.

Mesoporous silica nanoparticles (MSNs) were synthesized in a three-necked round-bottom flask to control the refluxing under a stream of N_2_ gas (Scheme 1), methodology adapted from Nandiyanto et al., (2009) [29]. In the first step, after dissolution of CTAB (0.1 g) in 30 mL of water in an ultrasonic bath for 1 min, we added octane (1.7 mL), styrene (12.7 µL) previously washed out with 2.5 mol L^−1^ sodium hydroxide solution (1:5, molar ratio) to remove the stabilizer, lysine (0.0215 g), TEOS (1.05 mL), and AIBA (0.0252 g). After 3 h of reaction under stirring at room temperature, the mixture was left for 12 h. After that, the MSNs were washed with ethanol and centrifuged. After that, the MSNs were dried in an electric oven at 60 °C overnight. Finally, the samples were calcinated for 3 h in a muffle at 500 °C at a heating rate of 1 °C min^−1^.

### 2.3. Synthesis of Chlorambucil Grafted MSNs

Firstly, the MSNs were coated by APTES following the Osseni methodology [30]. Then, 30 mg of MSNs was dispersed in 80 mL of isopropyl alcohol using an ultrasound bath for 2 h. After that, we added 7.5 mL of water, 8.9 mL of ammonium hydroxide, and 25 μL of TEOS. The solution was stirred at 40 °C for 2 h. After that, an excess of APTES (about 100 μL) was added to completely cover the MSN surface. The solution was kept under stirring at 40 °C for 1 h, then centrifugated at 6000 rpm for 30 min. The solid was washed with isopropyl alcohol three times, and finally the MSNs coated by APTES (MSNs@NH_2_) were dried in an oven at 50 °C overnight.

In the second step, the CLB was bonded on the MSNs@NH_2_ surface by dispersing 145 mg of MSNs@NH_2_ in 29 mL of an anhydrous ethanol solution of CLB (5 mg mL^−1^). The reaction was conducted under stirring at 40 °C for 24 h and, finally, MSNs@NH_2_-CLB was recovered by centrifugation at 6000 rpm for 30 min, washed with ethanol three times, and dried in a desiccator at room temperature.

### 2.4. Ninhydrin Assay (2,2-Dihydroxyindane-1,3-dione)

The detection of free NH_2_ groups on the surface of MSNs@NH_2_ was qualitatively determined using a ninhydrin assay. This substance reacts with primary amines to form a blue or purple complex known as Ruhemann’s purple, which absorbs in the visible region (570 nm). For the analysis, the nanoparticles were placed in a tube and dispersed in a solution containing 2 mL of ninhydrin (0.25% in acetone) and heated to 80–100 °C for 10 s.

### 2.5. Particle Size, Size Distribution

The particle size and polydispersity index (size distribution) were evaluated by dynamic light scattering (DLS). It was performed using a Zetasizer Nano-ZS model (Malvern Instruments, Malvern, UK). A polystyrene cuvette DTS0012 model from Malvern Instruments was used. The analysis was performed at 25 °C, in triplicate, using a suspension of nanoparticles, previously dispersed (24 h at 700 rpm) in water (0.01%, *w*/*v*).

### 2.6. Scanning Electron Microscopy—Field Emission Gun (SEM-FEG)

Intending to characterize the nanoparticle morphology, SEM-FEG microscopy was conducted using a JMS-6701F model from JEOL Ltd. (Akishima City, Tokyo) Samples were dispersed through the carbon surface of a metal disc (stub) after being previously dispersed in isopropanol (0.01% conc. of dispersed material).

### 2.7. Transmission Electron Microscopy (TEM)

Particle shape and size were examined by TEM using a JEM-1400 model from JEOL Ltd., with tungsten filaments, and operating from 40 to 120 kV. The samples were dispersed in isopropanol (0.01% conc. of dispersed material) and sonicated for 8 min, then 7 μL of the dispersed material was placed in a 400-mesh copper grid.

### 2.8. Fourier Transform Infrared Spectroscopy (FTIR)

The FTIR spectra were obtained by a Nicolet iS5 model from Thermo Fisher Scientific (Waltham, MA, USA), ranging from 4000 to 400 cm^−1^, with 4 cm^−1^ resolution and 100 scans min^−1^, and diluted in KBr pellets with 1% (*w*/*w*) final concentration.

### 2.9. Detecting Free Amino Groups on MSN@NH_2_-CLB Nanoparticle Surface

MSN@NH_2_-CLB nanoparticles were subjected to the ninhydrin assay to verify whether CLB had occupied all the NH_2_ groups on the MSNs@NH_2_ nanoparticle surface (Figure S5). A mass of 100 mg of MSN@NH_2_-CLB was suspended in water and in a pH 5.3 buffer solution, 2 mL of ninhydrin solution was added. A pH 5.3 solution simulates a tumor environment [31] and facilitates CLB release from the MSN@NH_2_-CLB surface. The assay was performed before and after CLB release from the nanoparticle surface.

### 2.10. CHN and Cl Elemental Analyses

The analyses were performed using a Perkin Elmer 2400 Series II analyzer from Perkin Elmer Instruments (São Paulo, Brazil). The samples were put through combustion under an atmosphere of pure oxygen; the resulting gasses were quantified by a thermal conductivity detector (TCD).

### 2.11. Thermogravimetric Analysis (TGA)

The TGA curves were acquired in a TGA Q50 V20,10 Build 36 model from TA Instruments (New Castle, DE, USA), in alumina crucibles with a heating rate of 20 °C min^−1^ from 0 to 900 °C under nitrogen purging (20 mL min^−1^) and air atmosphere (60 mL min^−1^).

### 2.12. Cytotoxicity Assay

The in vitro cytotoxicity of CLB and chlorambucil functionalized nanoparticles against adenocarcinoma human lung cancer cells (A549) and mouse colon cancer cells (CT26WT) were assessed by the MTT assay. [32] The cell line NIH-3T3 (normal murine fibroblast) was also evaluated to calculate the selectivity index (SI) of each substance. The assay was made by the Laboratory of Nanobiotechnology at the Genetic and Morphology Department of the Institute of Biology, University of Brasília, Brazil. The cytotoxic effect was evaluated by measuring the cell viability using 3-(4,5-dimethylthiazol-2-yl)-2,5-diphenyltetrazolium bromide (MTT). Briefly, the cell lines were cultured at 37 °C and 5% CO_2_ atmosphere and seeded in 96-well plates at a density of 3 × 10^3^ cells/well. The plates were incubated at 37 °C for 24 h. Then, the cells were incubated with various concentrations (0.625 to 100 μmol L^−1^) of CLB and CLB-functionalized nanoparticles. After 48 h, MTT solution in PBS was added and the plates were incubated for another 2.5 h at 37 °C and 5% CO_2_ atmosphere. After that, the medium containing MTT was removed and DMSO was added to each well to dissolve the MTT formazan crystals. Finally, the absorbance of the formazan solution was measured at 595 nm (Multiskan FC spectrometer from Thermo Scientific). The experiment was performed in triplicate and the mean ± standard deviation was reported.

The concentration that inhibits 50% of cell growth (IC_50_) was calculated by the non-linear regression curve from the cell viability values obtained in the MTT assay. To calculate the selectivity index (SI), the ratio between the IC_50_ observed for NIH-3T3 (normal cells) and the IC_50_ observed for CT26WT and A549 (cancer cells) [33] was obtained.

## 3. Results and Discussion

### 3.1. Nanoparticle Synthesis and Characterization

In recent years, mesoporous silica nanoparticles (MSNs) have emerged as a promising platform for targeted delivery of anticancer drugs and theragnostic applications. This is due to their unique ability to carry drugs both within their porous structure and on their functionalized surface.

It is stablished that nanoparticles with diameters smaller than 50 nm exhibit an excellent cellular and tissue permeability, which enhance the inhibition of cell proliferation [34]. However, particles smaller than 20 nm are cleared from the bloodstream by the lymphatic system [34,35]. Therefore, this study aimed to synthesize nanoparticles with a size between 20 and 50 nm as a way to enhance their cytotoxic effect against cancer cells.

In 2009, a methodology to obtain MSNs with a diameter of 45 nm was developed [17]. In our work, that methodology was modified. The temperature was reduced to 25 °C and the surfactant was solubilized in an ultrasonic bath before its use. With these changes, MSNs with a size of 30 ± 0.04 nm were synthetized (Figure S1).

The synthesis conditions used in our work allow for the control of two crucial factors: micelle stability and silica polycondensation rate. By enhancing the stability of micelles during formation, we prevent them from collapsing prematurely [21]. This ensures the formation of smaller and more consistent nanoparticles in terms of size distribution. Additionally, slowing down the rate of silica monomer condensation allows for more controlled growth of the nanoparticles. This controlled growth translates to a smaller final size, offering greater control over the final properties of the nanoparticles [21].

**Figure 2:**
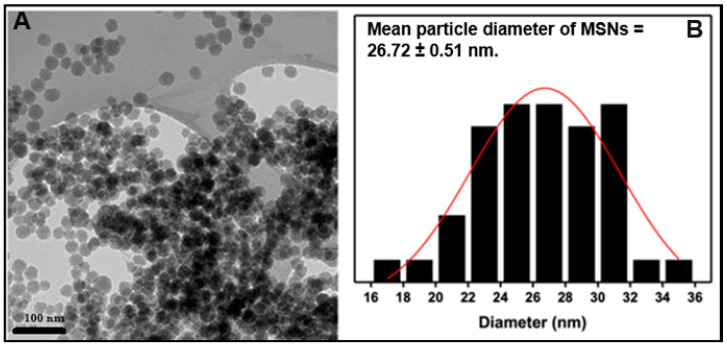
TEM microphotograph of MSNs (**A**) and their size distribution (**B**).

Nanoparticle morphology plays a critical role in their ability to penetrate cells and tissues. Spherical nanoparticles exhibit better circulation times in the bloodstream but may struggle to squeeze through tight spaces within tissues. Conversely, rod-shaped nanoparticles can efficiently infiltrate tissues but might be rapidly cleared from the bloodstream. By fine-tuning nanoparticle morphology, we can optimize their ability to reach specific targets within the body. This tailored design holds immense potential for targeted drug delivery, improving treatment efficacy and reducing side effects. [20]. Figure 2 shows TEM microphotographs of MSNs synthetized in this work. One can see spherical and porous particles with a size of 30 ± 0.04 (Figure 2B) (measured using ImageJ software, 1.53t). The size determined by this methodology corroborates the data obtained by DLS analysis (Figure S1).

### 3.2. Preparing MSN Surfaces for Functionalization

Nanoparticles alone struggle to deliver drugs specifically. Functional groups anchored to their surfaces act like docking stations, preparing the surface for functionalization. This targeted attachment allows drugs to hitch a ride straight to diseased cells, minimizing harm to healthy tissue [36]. In this work, the surfaces of MSNs were recovered with APTES, a compound with -NH_2_ functional groups that was used for the functionalization of MSNs with CLB.

After coating MSNs with APTES, we obtained the MSNs@NH_2_ complex. The presence of amine groups (-NH_2_) anchored to the MSN surface was confirmed using the ninhydrin test [36]. Primary amines react with ninhydrin to form a characteristic blue complex, indicating their presence [36]. MSNs alone produce the opposite result due to the absence of -NH_2_ groups.

**Figure 3:**
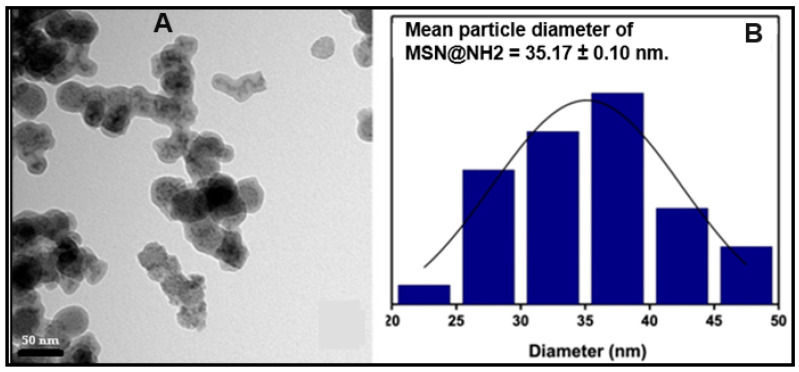
TEM microphotograph of MSN@NH_2_ (**A**) and their size distribution (**B**) observed by DLS.

Figure 3A shows MSNs@NH_2_ with a round morphology and a gray color around the nanoparticle core. This gray color likely arises from the presence of the primary amino groups on the surface [37]. Additionally, scanning electron microscopy (SEM-FEG) micrographs (Figure S2) confirm the complete coverage of the MSN surface with APTES.

Image analysis determined a particle size of 47.90 ± 0.66 nm, indicating an increase in size due to the APTES coating. Dynamic light scattering (DLS) measurements showed a mean particle size of 35.17 ± 1.0 nm for MSNs@NH_2_ with a size distribution (polydispersity index) of 0.090 (Figure 3B). Notably, both analyses revealed mean particle diameters below 50 nm.

### 3.3. MSNs@NH_2_-CLB Characterization

MSNs@NH_2_ were further functionalized with CLB. The primary amines present on the MSNs@NH_2_ surface readily react with the carboxyl groups of CLB, forming functionalized nanoparticles (MSNs@NH_2_-CLB). Fourier transform infrared spectroscopy (FTIR) confirmed the successful functionalization. Table 1 summarizes the absorption and stretching bands observed in the FTIR spectra of MSNs, MSNs@NH_2_, and MSNs@NH_2_-CLB (Figure S3). Bands at approximately 3400 cm^−1^ and 1635 cm^−1^ are attributed to the stretching (ν O-H) and bending (δ H-O-H) vibrations of silanol groups on the silica surface and water molecules adsorbed onto the nanoparticles [38,39].

In the regions of 1080 and 800 cm^−1^ can be observed intense bands characteristic of silica, which can be attributed to the asymmetric and symmetric stretching of the siloxane group (υ Si-O-Si), respectively. At 970 cm^−1^, a band corresponding to the asymmetric vibration of free silanol groups on the surface (ᵹ Si-OH) is observed [39].

The bands at 2922 and 2854 cm^−1^ observed on the spectrum of MSN@NH_2_ were attributed to the stretching vibration (υ C-H), a characteristic group of APTES added to the surface, confirming the coating and functionalization of MSNs [39].

The MSN@NH_2_-CLB spectrum shows an intense band at 1515 cm^−1^, which was not observed for MSN@NH_2_ (Figure S3). This fact confirms the presence of CLB anchored on the MSN@NH_2_ surface. This band can be attributed to N-H bending, characteristic of ammonium salts, which exhibit intense N-H bending bands in the 1610–1500 cm^−1^ region [28].

The peak at 1710 cm^−1^, characteristic of the carboxylic acid C=O bond in CLB, disappears. Instead, a new peak appears at 1633 cm^−1^. This shift indicates the conversion of the carboxylic acid group to a carboxylate ion. Carboxylate groups typically show strong vibrations around 1600 cm^−1^, confirming this transformation [28]. The ionic bonding of carboxyl groups from CLB to amino groups on MSN@NH_2_ forms ammonium carboxylate. This salt is formed because carboxylic acids do not undergo nucleophilic acyl substitution reactions with amines. Since the carboxylic acid has a lower pK_a_ than the amine, the carboxylic acid immediately donates a proton to the amine when both substances are mixed [40].

It was verified that CLB reacted with all NH_2_ groups on the MSN@NH_2_ surface. The ninhydrin test suggests successful CLB attachment to the NH_2_ groups on the MSN@NH_2_ surface. Suspensions of both MSN@NH_2_ (in water) and MSN@NH_2_-CLB (in buffer solution) exhibited the characteristic blue color associated with the ninhydrin–primary amine complex. However, the color was not observed in the aqueous suspension of MSN@NH_2_-CLB, which suggests that CLB is bound to all the NH_2_ groups present on the MSN@NH_2_ surface. This fact confirms that the conditions used to attach CLB to MSNs@NH_2_ were adequate.

### 3.4. Elemental Analysis for Chlorine to Quantify CLB Loaded in Nanoparticles

Table 2 shows the chlorine elemental analysis performed to quantify the CLB loaded to the MSN@NH_2_ surface. One gram of each nanoparticle was used for the analysis. As expected, no chlorine was detected in MSNs@NH_2_. The chlorine content measured in CLB was 23.25 ± 0.05%, which shows no statistically significant difference from the theoretical value of 23.27%. (Here, 23.25 ± 0.05 encompasses 23.27%).

The chlorine content in MSN@NH_2_-CLB was 1.63 ± 0.01%, corresponding to a loaded CLB mass of approximately 70.0 mg. This chlorine detection confirms successful CLB attachment to the MSN@NH_2_ surface. Importantly, it also indicates that the chemically unstable CLB molecule remained intact, preserving the active nitrogen mustard group [41].

### 3.5. Thermogravimetric Analysis

Thermogravimetric analysis (TGA) of MSNs (Figure S4) revealed two key thermal events. The first, below 300 °C, likely corresponds to the desorption of physically adsorbed water. The second event, above 650 °C, can be attributed to the vitreous transition of silica, as reported previously [42]. MSN@NH_2_ and MSN@NH_2_-CLB also exhibited mass losses in this high-temperature range (2.2% and 3.8%, respectively), suggesting increased hydrophobicity of the material upon CLB incorporation.

Mass loss between 100 and 800 °C typically indicates the decomposition of organic matter bound to silica [43,44]. In this range, MSN@NH_2_ displayed a 9.8% mass loss, attributed to the decomposition of molecules associated with the nanoparticle coating (APTES). Notably, MSN@NH_2_-CLB exhibited a higher mass loss (16.8%), indicating the presence of additional organic material—the CLB molecules.

The amount of CLB loaded was also estimated by the TGA data. The difference in mass loss between MSN@NH_2_ and MSN@NH_2_-CLB was approximately 7%, corresponding to 70.0 mg of CLB per gram of MSN@NH_2_-CLB. This confirms the result obtained by the stoichiometric chlorine analysis.

This loading capacity corresponds to approximately 38% of the amount reported by Karthik et al. [45], who achieved a CLB loading of 190 mg/g using a CLB-Quinoline conjugate. It is important to note that their work likely incorporated CLB throughout the silica matrix, utilizing pore functionalities as additional binding sites. In contrast, our method targets only the outer surface of the nanoparticles, resulting in a lower CLB loading. Nonetheless, the fact is that CLB being anchored to the surface facilitates CLB release; however, more studies must be made in order to confirm this statement.

### 3.6. Cytotoxic Effect

To assess whether the addition of CLB to MSNs would enhance their cytotoxic effect, the cytotoxicity of CLB and MSN@NH_2_-CLB were evaluated (Table 3 and Figure S6).

The selectivity index (SI) of both nanoparticles was also calculated. SI is an important parameter that indicates how much a cytotoxic substance impact tumor cells keeping the integrity of normal cells. As a rule, SI values greater than two are considered acceptable [33]. CLB exhibited a good cytotoxicity (IC_50_ 62.39 μmol L^−1^) against CT26WT cells, with an SI of 1.37. In the same way, the IC_50_ value of CLB against A549 cells was 115.2 μmol L^−1^, with an SI of 0.74, indicating that the drug is more cytotoxic towards normal cells than towards this line of carcinogenic cells.

This study showed that MSN@NH_2_-CLB was more effective against cancer cells. Its concentration needed to inhibit 50% of cell growth (IC_50_) was much lower for cancer cell lines (A549 at 17.71 μmol L^−1^ and CT26WT at 27.67 μmol L^−1^) compared to normal cells (NIH-3T3). This is reflected in the selectivity index, which was significantly higher for cancer cells (6.54 for A549 and 4.19 for CT26WT). Higher SI indicates a greater preference for killing cancer cells over healthy ones.

It was reported the cytotoxicity of various silica-CLB nanomaterials against MDA-MB-231 cell, a kind of human breast cancer cell [28]. Their findings show that nanomaterials with functionalized surfaces exhibit a stronger dose-dependent cytotoxic effect compared to those materials with CLB loaded directly to the surface. In simpler terms, the addition of functional groups made the silica–CLB more toxic to cancer cells as the dose increased.

In this research, the cytotoxicity assay demonstrated that CLB when anchored to APTES-coated mesoporous silica nanoparticles exhibits significantly greater cytotoxic activity towards carcinogenic cells than unbound chlorambucil. MSN@NH_2_-CLB exhibited 8.84 times greater selectivity towards the A549 cell line than unbound CLB in nanoparticles. Similarly, the selectivity of MSN@NH_2_-CLB towards the CT26WT cell line was three times greater than that of unbound CLB in nanoparticles. The higher the selectivity index of a substance, the lower its cytotoxic effect on normal cells [33,46]. This is extremely important for chemotherapy drugs, which in most cases, produce adverse effects like hair loss, leukemia, skin diseases, vision problems, and others, due to their low selectivity, causing cytotoxic effects in both cancer cells and normal cells.

## 4. Conclusions

In this study, we successfully synthesized mesoporous silica nanoparticles (MSNs) with a diameter of about 30 nm, suitable for targeted drug delivery applications. The employed methodology yielded MSNs within the desired size range (20–50 nm) for enhanced cellular uptake and improved therapeutic efficacy. The presence of CLB on the surface of the nanoparticles (MSN@NH2-CLB) was confirmed using various techniques, including FT-IR, TGA, and elemental analysis. MSN@NH2-CLB exhibited significantly higher cytotoxicity against colon carcinoma (CT26WT) and lung adenocarcinoma (A549) cells compared to free CLB. This indicates the potential of these nanoparticles for targeted cancer therapy. The cytotoxicity assay results demonstrated a higher selectivity index for MSN@NH2-CLB compared to free CLB, indicating a preferential cytotoxic effect on cancer cells over healthy cells. This is a crucial factor for minimizing side effects during chemotherapy. These findings suggest that CLB-functionalized MSNs hold promise as a drug delivery platform for targeted cancer therapy. However, further studies are needed to explore their performance in vivo and optimize their therapeutic potential.

## Figures and Tables

**Table 1:** Assignments of FTIR bands for MSNs, MSN@NH_2_, and MSN@NH_2_-CLB.

Nanoparticles	Assignments (cm^−1^)								
	υ(H-O-H)	υ(C-H)	υ(C=O)	υ(O-H)	υ(N-H)	υ(Si-O)	υ(Si-OH)	υ(Si-O-Si)	υ(Si-O)
MSN	~3400	-	-	1640	-	1100	970	814	465
MSN@NH_2_	~3400	2949	-	1635	1523	1112	960	802	467
MSN@NH_2_-CLB	~3400	2943	1620	-	1515	1110	962	804	469

**Table 2:** Elemental analysis for chlorine in CLB, MSN@NH_2,_ and MSN@NH_2_-CLB.

Mass	Nanoparticle		
	CLB	MSN@NH_2_	MSN@NH_2_-CLB
Chlorine (%)	23.25 ± 0.05	0	1.63 ± 0.01
CLB (mg)	1000 ± 1.23	0	70,00 ± 0.05

*n* = 3, mass used for the analysis 1.00 g.

**Table 3:** IC_50_ values and selectivity index of chlorambucil and CLB-functionalized nanoparticles (MSN@NH_2_-CLB) on human lung adenocarcinoma cells (A549) and CT26WT cells (colon carcinoma) as well as NIH-3T3 cell (normal cells) after 48 h of treatment.

Substances	Cell Line			Selectivity Index	
	NIH-3T3	A549	CT26WT	A549	CT26WT
Chlorambucil	85.50 ± 1.18	115.2 ± 1.21	62.39 ± 1.52	0.74	1.37
MSN@NH_2_-CLB	115.9 ± 1.13	17.71 ± 1.26	27.67 ± 1.17	6.54	4.19

## Data Availability

The data presented in this study are available on request from the corresponding author.

## Supplementary Materials

The following supporting information can be downloaded at: https://www.mdpi.com/article/10.3390/pharmaceutics16081086/s1, Figure S1: DLS Histogram of the MSN; Figure S2: SEM-FEG imagens and size-distribution histograms of the MSN@NH2 sample; Figure S3: FT-IR spectra of the samples MSN, MSN@NH2, and MSN@NH2@CLB using KBr pellets; Figure S4: Thermogravimetric Analysis (TGA) of MSNs, MSN@NH2, and MSN@NH2-CLB; Figure S5: Primary amine identification assay using ninhydrin solution. (a) SiO_2_@NH2, (b) SiO_2_@NH2-CLB, (c) SiO_2_@NH2-CLB after CLB release in acidic medium; Figure S6: MTT assay: graph depicting the cell viability percentage as a function of varying CLB concentration: (a) NIH-3T3; (b) CT26 WT and (c) A549 cell lines.
