# Supplementary material for: Synthesis, Characterization, and Cytotoxicity Evaluation of Chlorambucil-Functionalized Mesoporous Silica Nanoparticles
Source: Pharmaceutics. 2024 Aug 19;16(8):1086. doi: 10.3390/pharmaceutics16081086 (PMC11359805; doi:10.3390/pharmaceutics16081086)
Supplement: Supplementary file 1 [file pharmaceutics-16-01086-s001.zip › pharmaceutics-3062442-supplementary.pdf]

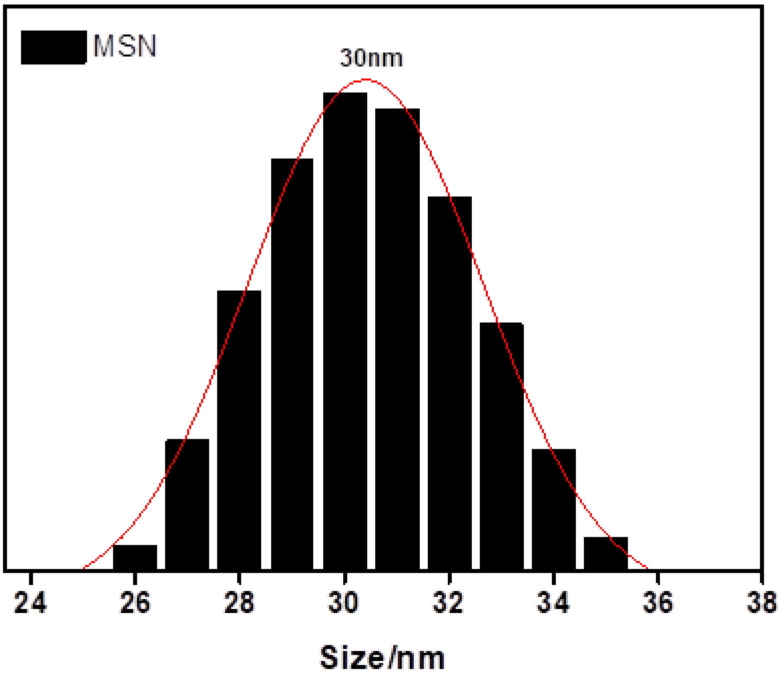

Figure S1. DLS Histogram of the MSN

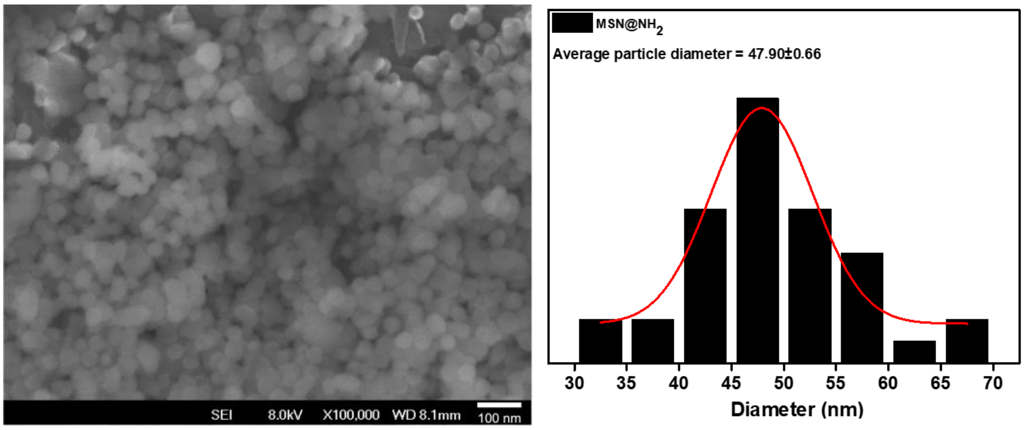

Figure S2. SEM-FEG imagens and size-distribution histograms of the MSN@NH2 sample.

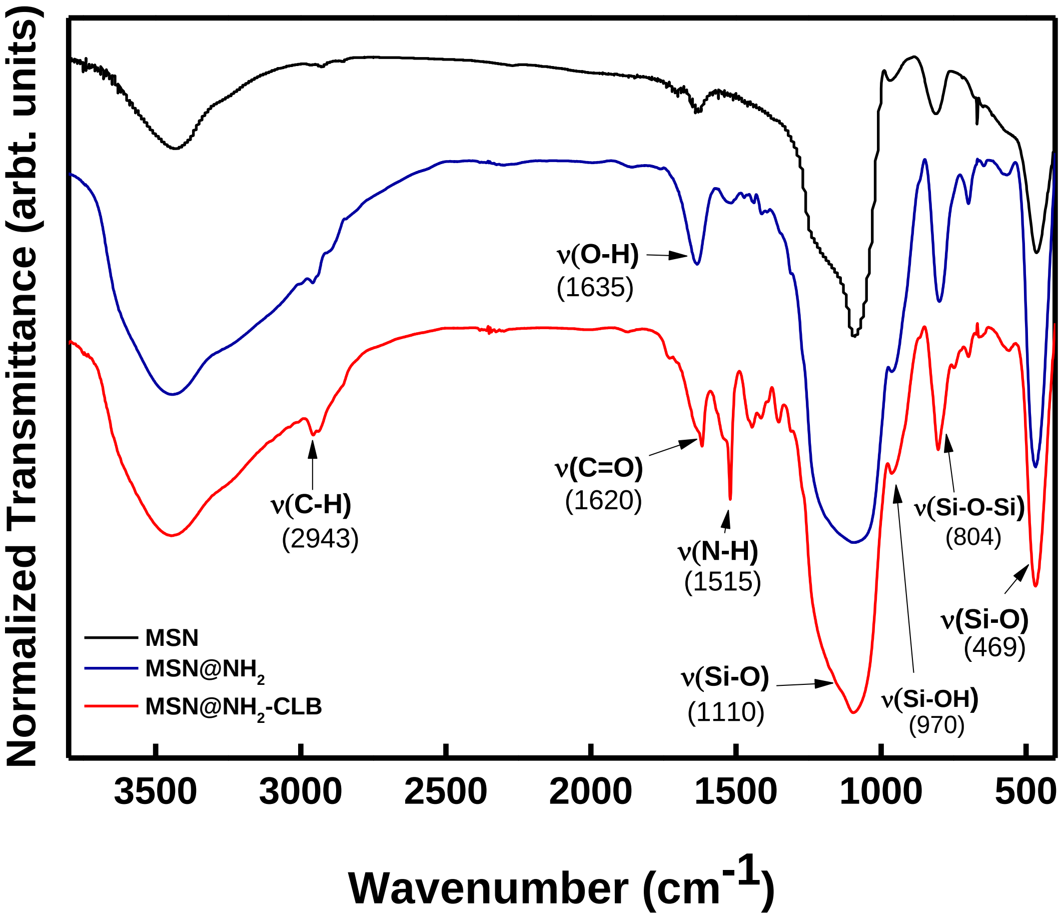

Figure S3. FT-IR spectra of the samples MSN, MSN@NH2, and MSN@NH2@CLB using KBr pellets.

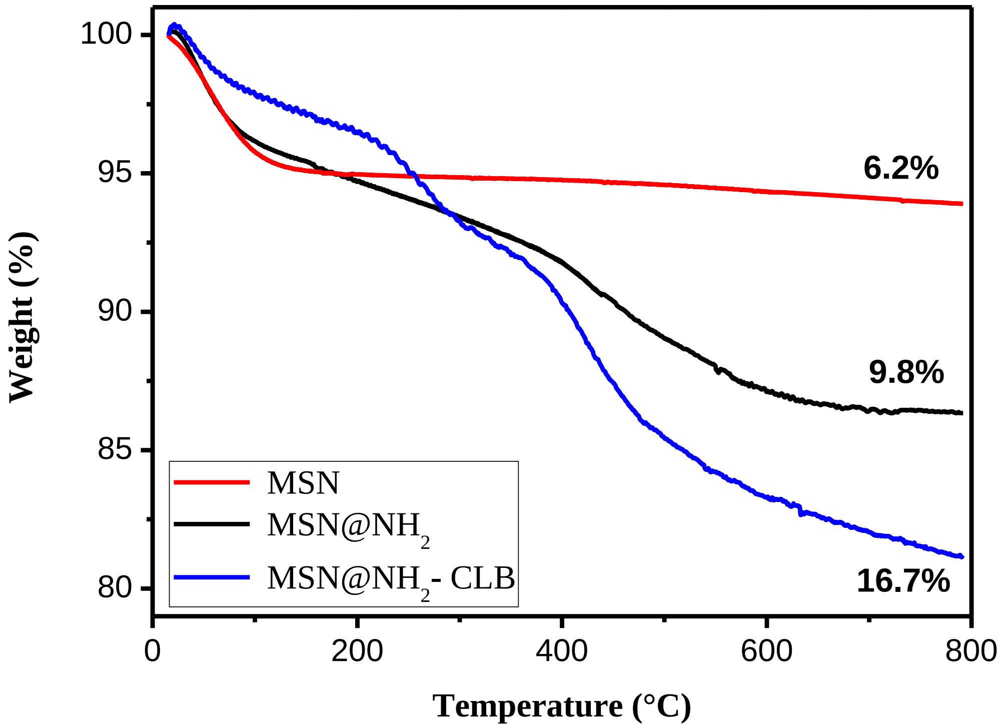

Figure S4. Thermogravimetric Analysis (TGA) of MSNs, MSN@NH2, and MSN@NH2-CLB

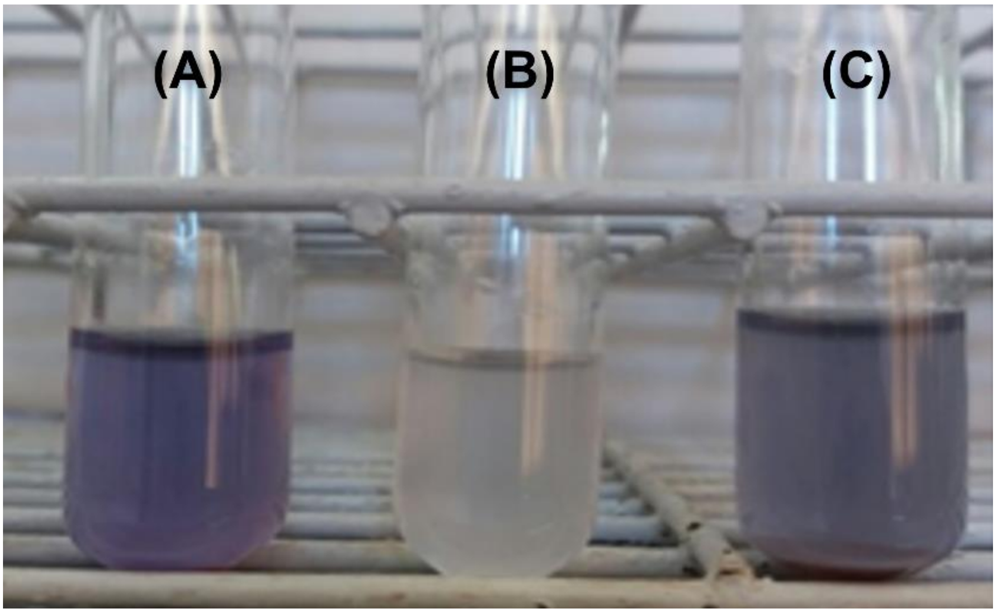

Figure S5. Primary amine identification assay using ninhydrin solution. (**A**) SiO2@NH2 (**B**) SiO2@NH2-CLB (**C**) SiO2@NH2-CLB after CLB release in acidic medium

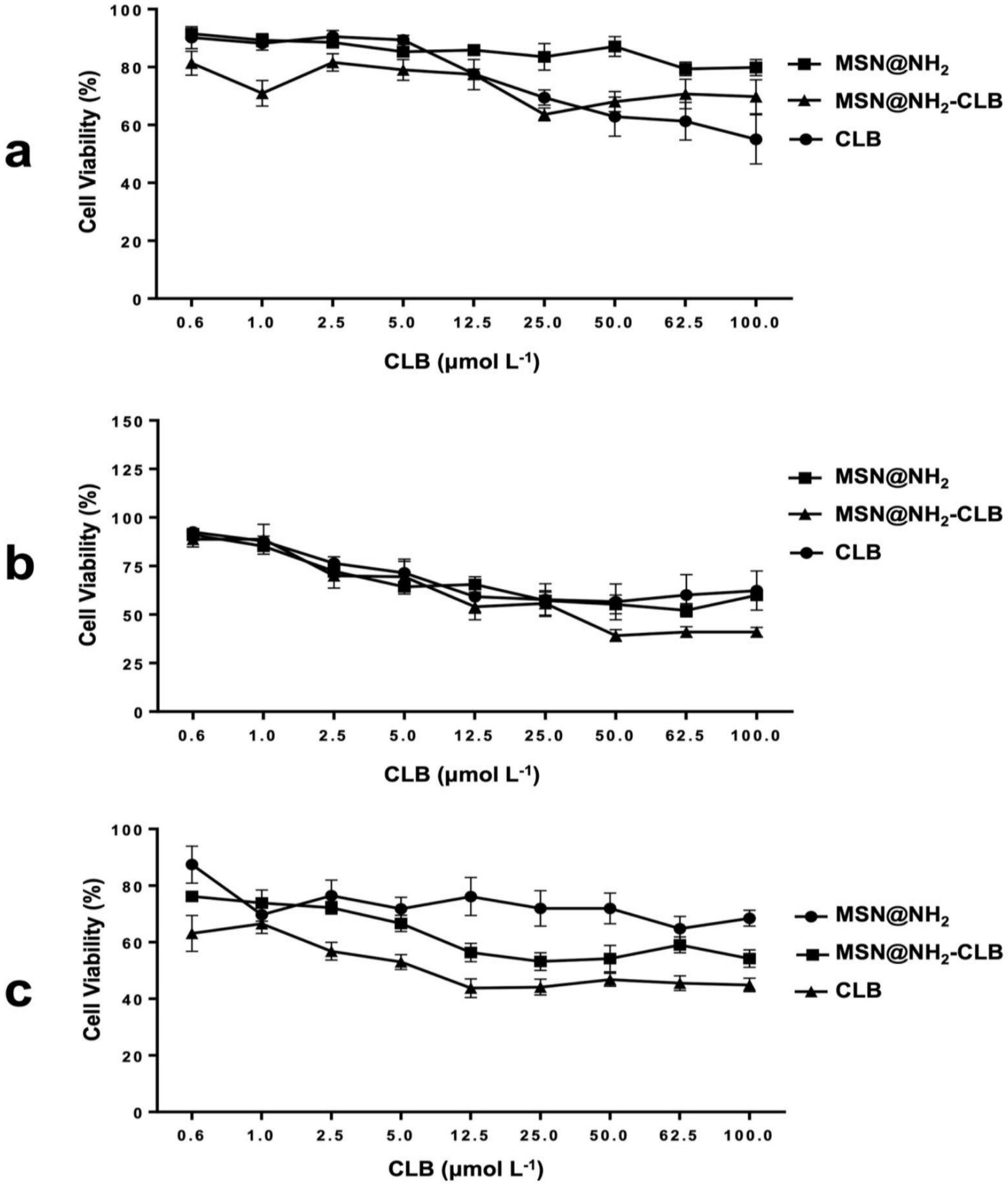

Figure S6. MTT assay: graph depicting the cell viability percentage as a function of varying CLB concentration: (**a**) NIH-3T3; (**b**) CT26 WT and (**c**) A549 cell lines.
